# Involvement of Mitochondria in Parkinson’s Disease

**DOI:** 10.3390/ijms242317027

**Published:** 2023-12-01

**Authors:** Chi-Jing Choong, Hideki Mochizuki

**Affiliations:** Department of Neurology, Osaka University Graduate School of Medicine, 2-2 Yamadaoka, Suita 565-0871, Osaka, Japan; choongcj@neurol.med.osaka-u.ac.jp

**Keywords:** Parkinson’s disease, mitochondria, Parkin, PINK1, extracellular mitochondria, mitochondria transplantation

## Abstract

Mitochondrial dysregulation, such as mitochondrial complex I deficiency, increased oxidative stress, perturbation of mitochondrial dynamics and mitophagy, has long been implicated in the pathogenesis of PD. Initiating from the observation that mitochondrial toxins cause PD-like symptoms and mitochondrial DNA mutations are associated with increased risk of PD, many mutated genes linked to familial forms of PD, including *PRKN*, *PINK1*, *DJ-1* and *SNCA*, have also been found to affect the mitochondrial features. Recent research has uncovered a much more complex involvement of mitochondria in PD. Disruption of mitochondrial quality control coupled with abnormal secretion of mitochondrial contents to dispose damaged organelles may play a role in the pathogenesis of PD. Furthermore, due to its bacterial ancestry, circulating mitochondrial DNAs can function as damage-associated molecular patterns eliciting inflammatory response. In this review, we summarize and discuss the connection between mitochondrial dysfunction and PD, highlighting the molecular triggers of the disease process, the intra- and extracellular roles of mitochondria in PD as well as the therapeutic potential of mitochondrial transplantation.

## 1. Introduction

Mitochondria are essential for cellular function due to their involvement in ATP production via oxidative phosphorylation, lipid metabolism assembly, regulation of homeostasis and programmed cell death [1]. As the main source of energy support, changes in mitochondrial functions have a large impact on cellular activities. It is widely recognized that mitochondrial dysfunction occurs in many neurodegenerative diseases as neurons are cells with high energy demand, and the nervous system is particularly sensitive to alterations in mitochondrial quality control mechanisms [2]. In Parkinson’s disease (PD), a progressive motor disorder characterized by selective dopaminergic neuronal degeneration in the substantia nigra (SN), mitochondrial dysfunction in the form of compromised complex I activity and bioenergetic capacity, increased oxidative stress and reduced resistance of stress have been observed [3,4]. High levels of mitochondria DNA (mtDNA) deletions are reported to be associated with respiratory chain deficiency and selective neuronal loss in aging and PD [5,6,7,8,9]. Moreover, the identification of genes including *PRKN*, *PINK1*, *DJ-1* and *SNCA* linked to familial variants of PD has revealed a common pathway involving mitochondrial quality control and dynamics [10,11,12,13]. All these have fueled research on the mitochondrial aspects of disease etiopathogenesis.

Recent studies have shown that mitochondrial content can also be found outside of cells, either as free mtDNA, functional or damaged mitochondria or within extracellular vesicles, where it could contribute to the etiology of PD [14,15,16,17]. Also, there has been a dramatic change to the perception of mitochondria being only the powerhouse of cell as it is now acknowledged that apart from being essential intracellularly, mitochondria can promote cellular repair when being transferred from healthy to damaged cells and hence exploration of the therapeutic use of mitochondrial transplantation [18,19,20].

In this review, we review studies reporting the link between mitochondria dysfunction and PD; the role of mitochondria, both intra and extracellularly, in PD; and how mitochondrial transplantation could serve as a potential therapeutic strategy to rescue tissue damage and degeneration in PD.

## 2. Origin of the Link of Mitochondria to PD

The first clue connecting mitochondrial dysfunction to PD came from the discovery of 1-methyl-4-phenyl-1,2,3,6-tetrahydropyridine (MPTP), a contaminant of synthetic heroin, which produced a severe, permanent parkinsonian syndrome responsive to levodopa treatment in a number of drug abusers in the 1980s. Autopsy findings in one case revealed that MPTP appears to destroy neuronal cells in the SN, an area of the brain that plays a major role in controlling movement, and this has initiated a renaissance of basic research in PD [21]. Studies on the mechanism of action of this compound and its effects enable us to understand its role in the nigrostriatal system. MPTP is not toxic itself but is a lipophilic compound that can cross the blood–brain barrier. In the brain, it is processed by monoamine oxidase B in glial cells to the toxic cation 1-methyl-4-phenylpyridinium (MPP+), which is selectively taken up by dopaminergic cells and inhibits mitochondrial respiration by blocking the mitochondrial complex I, leading to a reduction in cellular ATP [22,23]. Although this appears to be a major step in blocking mitochondrial function, studies also demonstrate that MPP+ can directly inhibit complexes III and IV of the electron transport chain [24]. The loss of cellular energy leads to the generation of reactive oxygen species including superoxide radicals, hydrogen peroxide, and hydroxyl radicals [25]. These findings implicate the involvement of mitochondrial dysfunction to PD pathogenesis and has led to increased concern regarding the environmental causes of this disease. Besides MPTP, there are several other complex I inhibitors commonly used as pesticides, like rotenone, fenazaquin, trichloroethylene, paraquat, tebunfenpyrad and fenpyroximate, of which exposure contributes to the pathogenesis of sporadic PD [26].

More importantly, mitochondrial complex I deficiency has been observed in the postmortem striata and substantia nigra of PD patients [3,27]. Mitochondrial complex I deficiency was also observed in the frontal cortex and in non-brain tissues such as platelets and skeletal muscle, suggesting a global reduction in mitochondrial complex I activity in PD [28,29,30]. Isolation of PD brain mitochondria revealed that complex I has oxidatively damaged subunits and is functionally impaired and misassembled [4].

These observations brought into question whether mitochondrial complex I deficiency is the cause of disease or rather a byproduct of sporadic PD. Recent work by Gonzalez-Rodriguez et al. discovered mitochondrial complex I dysfunction to be sufficient to cause progressive parkinsonism. Genetically engineered mice lacking the gene encoding a complex I subunit called NDUFS2 in dopaminergic neurons showed a nigrostriatal axonal deficit accompanied by motor learning and fine motor deficits [31].

## 3. Genes Particularly Associated with Mitochondrial Dysfunction in PD

Genetic studies focusing on monogenic forms of PD provided further support for the involvement of mitochondrial dysfunction in the disease. Pathogenic mutations in *PINK1* (PARK6), *PRKN* (PARK2), *DJ-1* (PARK7), *SNCA* (PARK1), *FBXO7* (PARK15), *CHCHD2* (PARK22) and *VPS13C* (PARK23) cause familial PD, and the majority of the proteins encoded are known to be involved in the mitochondrial quality control system and, in particular, mitophagy (Table 1) [13]. A recent study combining the availability of large-scale genome-wide association data in PD cases with current statistical tools, such as polygenic risk scoring and Mendelian randomization, further found potential causal association of 14 novel mitochondrial-function-associated genes with PD risk, and these genes are not only involved in mitophagy but implicate distinct mitochondrial processes [32].

### 3.1. Parkin

Parkin is a protein encoded by the PARK2 *PRKN* gene, through which the loss-of-function mutation causes autosomal recessive juvenile parkinsonism characterized by selective dopaminergic neuronal death and absence of Lewy bodies [10]. Parkin, an E3 ubiquitin ligase, ubiquitinates and targets protein substrates for degradation via the ubiquitin proteasome system [33]. In the setting of mitophagy, parkin tags damaged mitochondria with ubiquitin to activate autophagic degradation. There are new theories on the role of parkin. A study described that parkin orchestrates mitophagy by coordinating mitochondrial lipid remodeling. Parkin induces the production of phosphatidic acid and its subsequent conversion to diacylglycerol, which requires mitochondrial ubiquitination and ubiquitin-binding autophagy receptors, NDP52 and optineurin. Hence, mitochondrial diacylglycerol production acts downstream of parkin to facilitate the local assembly of autophagosomes for disposal of ubiquitinated mitochondria [34]. Another study reported that parkin monomers are not recruited to abnormal mitochondria, and parkin precipitates on mitochondria via self-aggregation and autoubiquitination. Parkin aggregates positively modulate mitophagy [35].

Parkin downregulation induced apoptotic cell death of human dopaminergic cells associated with caspase activation, accompanied by the accumulation of oxidative dopamine metabolites [36]. Parkin-deficient flies and mice exhibited mitochondrial impairment and oxidative damage [37,38,39]. As the animal models could not fully recapitulate the pathophysiology of human PARK2, a study group generated induced pluripotent stem cells (iPSCs) from PARK2 patients. Neurons derived from PARK2 iPSCs exhibited aberrant mitochondrial morphology and impaired mitochondrial homeostasis associated with increased oxidative stress [40]. PARIS (ZNF746), a KRAB and zinc finger protein, has been identified as a parkin-interacting substrate, and its level is regulated by the ubiquitin proteasome system via binding to and ubiquitination by parkin. Accumulation of PARIS can be observed in models of parkin inactivation and in the human PD brain. PARIS represses the expression of the transcriptional coactivator, peroxisome proliferator-activated receptor gamma coactivator-1 alpha (PGC-1α), which positively regulates the expression of genes required for mitochondrial biogenesis and the cell’s antioxidant responses. Conditional knockout of parkin in adult mice led to PARIS-dependent transcription repression of PGC-1α, declines in mitochondrial mass and respiration and eventually progressive loss of dopaminergic neurons [41,42]. Interestingly, certain parkin modifications can be pathologic. Parkin has been shown to be S-nitrosylated in vitro, as well as in vivo in a mouse model of PD and in the brains of patients with idiopathic PD. S-nitrosylation of parkin compromises its E3 activity and protective function [43,44]. Parkin has also been reported to be modified covalently by dopamine in the SN, but no other regions of the human brain. The modification increases parkin insolubility and inactivates its E3 ubiquitin ligase function in the brains of idiopathic PD patients [45]. Parkin sulfhydration, on the contrary, enhances its catalytic activity and is prominently depleted in the brains of idiopathic PD patients compared to those of control subjects, implying that a decrease in such modification of parkin may be unfavorable [46].

### 3.2. PINK1

PTEN-induced putative kinase 1 (PINK1) is a mitochondrial serine/threonine-protein kinase that is encoded by *PINK1* gene. Parkin and PINK1 work together in the mitochondrial quality control pathway, regulating mitochondrial morphology, dynamics and clearance. In healthy mitochondria, there is very low or undetectable level of PINK1 on the outer mitochondrial membrane as the protein is rapidly imported into the mitochondrial inner membrane, cleaved by presenilin-associated rhomboid-like protein (PARL) and degraded. Upon mitochondrial depolarization, full-length PINK1 is stabilized on the outer mitochondrial membrane, where it phosphorylates ubiquitin on the surface of the mitochondria [47,48,49,50]. An early model of PINK1/parkin mitophagy depicts that PINK1 acts upstream of parkin with a minor initiator role whose main function is to phosphorylate ubiquitin to activate the ubiquitin ligase parkin. Parkin then forms ubiquitin chains on the mitochondrial outer membrane proteins that help recruit autophagy receptors [51,52]. A current model suggests that PINK1 has a more direct role in initiating mitophagy at a low level by generating phospho-ubiquitin on mitochondria to recruit autophagy receptors, without involving parkin. To trigger robust and rapid mitophagy, parkin activation is required to amplify the PINK1-generated phospho-ubiquitin signal [53].

Loss-of-function mutations in the *PINK1* gene lead to autosomal recessive forms of PD [11]. When expressed in cultured cells, PINK1 mutants lose the ability to prevent mitochondrial permeability transition pore opening and cytochrome c release, caspase 3 activation and apoptotic cell death [54]. In dopaminergic neurons, overexpression of PD-linked PINK1 mutations upregulates TH and DA levels and increases their vulnerability to oxidative stress [55]. In the Drosophila model, pink1 mutants undergo apoptotic muscle degeneration and fragmentation of mitochondrial cristae, are sensitive to multiple stresses and have reduced lifespan and ATP levels [56]. Loss of Drosophila parkin shows phenotypes similar to the loss of pink1 function, strengthening the notion that pink1 and parkin function, at least in part, in the same pathway [38,39].

Confocal imaging of mitochondria function and morphology indicated that SN dopaminergic neuronal cultures from PINK1 knockout mice exhibited a reduction in mitochondrial membrane potential, mitochondrial fragmentation as well as elevated oxidative stress [57]. Electron microscopy analysis on the striatum of PINK1 knockout mice revealed no evident ultrastructural defects in mitochondria but an increased number of larger mitochondria, in line with the role of PINK1 in regulating mitochondrial fission. Furthermore, mitochondrial functional defect and increased sensitivity to oxidative stress were observed [58,59]. Defects in PINK1 also impair the oxidative stress-induced antioxidant heme oxygenase-1 expression [60]. Furthermore, a transgenic mouse model that has a combined etiology of PINK1 deletion and mitochondrial stress caused by the conditional expression of mitochondrial unfolded ornithine transcarbamylase with the TH promoter showed accelerated neurodegeneration as compared to the transgenic mice with wild-type background and also no responsiveness to L-dopa [61].

Alpha synuclein (αSyn) is a key protein involved in PD pathogenesis. There is substantial evidence proving that αSyn-induced neurotoxicity is exacerbated by the loss of PINK1. PINK1 knockdown aggravated αSyn-induced aggregation and cell death in a cell culture model of synucleinopathy. Similarly, loss of PINK1 accelerated the αSyn-induced neuropathology and neurodegenerative phenotypes in an in vivo mouse model [62]. Double-mutant mice with PINK1 ablation and αSyn mutant A53T overexpression showed more severe phenotypes, including high mortality and decreased spontaneous motor activities from an early age, compared to single-mutant mice. A number of double-mutant mice displayed progressive paralysis at old age and protein aggregates with composition reminiscent of the neuronal Lewy pathology in human PD. Moreover, transcriptional dysregulations reflecting anomalies in mitochondria, autophagy, microtubular network, synapse, ubiquitination and DNA damage repair were only observed in double-mutant mice [63].

Conversely, studies have shown that PINK1 confers a protective effect against αSyn-induced cytotoxicity. PINK1 interacts with αSyn through its kinase domain in the cytoplasm. This interaction prevents αSyn localization to mitochondria and induces αSyn degradation by autophagy, thereby abrogating αSyn-induced neurotoxicity [64]. In a Drosophila model of PD, pink1 overexpression counteracted αSyn-induced phenotypes including premature loss of climbing ability, disruption of ommatidial array and developmental eye defects [65]. A subsequent study reported a synergistic effect of pink1 and αSyn coexpression in the dopaminergic neurons in extending the lifespan and healthspan of Drosophila [66].

### 3.3. Fbxo7

PINK1 and parkin engage in the molecular pathway for the clearance of damaged mitochondria. Burchell et al. [67] found that a third protein, F-box protein 7 (Fbxo7), encoded by PARK15, mutations of which cause early-onset autosomal recessive PD with pyramidal tract signs, also functions in this pathway [68]. Fbxo7 shares a role in mitochondrial maintenance through direct interaction with PINK1 and parkin. It facilitates parkin recruitment to damaged mitochondria, mitofusin 1 ubiquitination and subsequent mitophagy. Three PD-causing mutations in *FBXO7* namely T22M, R378G and R498X, interfered with this function in both flies and mammalian cells. In Drosophila, ectopic overexpression of Fbxo7 rescued loss of parkin but not loss of pink1, indicating that Fbxo7 functions downstream of pink1 [67,69]. Fbxo7 can promote the ubiquitination of mitochondrial proteins to regulate basal mitophagy in a pink1- and parkin-independent manner. The ubiquitinated mitochondrial proteins serve as the substrate for pink1-mediated phosphorylation to initiate stress-induced mitophagy [69]. However, a study argues against the role for Fbxo7 in parkin-dependent mitophagy as absence of Fbxo7 causes no obvious alterations in mitochondria [70].

### 3.4. Alpha Synuclein

αSyn is a presynaptic neuronal protein genetically and neuropathologically linked to PD. Missense mutations and multiplications in the *SNCA* gene encoding αSyn cause familial forms of PD. αSyn can be converted from a monomeric state into oligomers and finally disease-related amyloid fibrils and is a major constituent of Lewy bodies, protein clumps that are the pathological hallmark of familial as well as sporadic PD. The linkage between αSyn and mitochondrial dysfunction has recently been identified. αSyn protein contains a mitochondrial targeting sequence at its N-terminus and is translocated to mitochondria and predominantly associated with the inner membrane. Accumulation of wild-type αSyn in the mitochondria of human dopaminergic neurons suppressed mitochondrial complex I activity and increased formation of reactive oxygen species. Mitochondria of the affected regions, substantia nigra and striatum, but not cerebellum from PD subjects, showed increased accumulation of αSyn and decreased complex I activity [71]. αSyn interacts with complex I, impairs its function, promotes production of reactive oxygen species and causes direct mitochondrial toxicity [71,72,73,74]. In vitro assay has shown that αSyn has an inhibitory function in membrane fusion. Direct interaction of αSyn with mitochondrial membranes can lead to mitochondrial fragmentation, which is eventually followed by a decline in respiration and neuronal death. This can be rescued by coexpression of PINK1, parkin or DJ-1 but not the PD-associated mutations [75,76]. Intracellular pH is reported to affect the cellular distribution of αSyn. Oxidative and metabolic stresses induce cytosolic acidification, which results in translocation of αSyn from the cytosol onto the mitochondrial outer membrane. Binding is likely facilitated by low pH-induced exposure of the mitochondria-specific lipid cardiolipin [77]. Moreover, intracellular seeding of αSyn occurs preferentially on membrane surfaces, especially at mitochondrial membranes. The mitochondrial lipid cardiolipin initiates rapid oligomerization of A53T αSyn, and cardiolipin was found to be sequestered within aggregating lipid-protein complexes. These aggregates interfere with complex I activity and augment mitochondrial reactive oxygen species generation. This subsequently hastens the oligomerization of A53T αSyn and causes mitochondrial membrane permeabilization and cell death. These processes were also observed in iPSC-derived neurons from PD patients with the A53T mutation [78]. Another study reported that overactivation of macroautophagy in primary cortical neurons that overexpressed mutant A53T αSyn leads to massive mitochondrial destruction and loss and eventually bioenergetic deficit and neuronal degeneration [79]. In an α-Synucleinopathy animal model, an increase in oxidative stress and mitochondrial complex I dysfunction accompanied neurodegeneration in the brain regions including substantia nigra pars compacta, striatum, hippocampus and olfactory bulb [80]. A mouse model deficient in *PLA2G6*, the causative gene for PARK14-linked autosomal recessive early-onset dystonia-parkinsonism, showed elevated expression of αSyn and pSyn granules being localized to mitochondria with degenerated inner membranes. In the neurons derived from brain tissue of patients with *PLA2G6*-associated neurodegeneration, small pSyn-positive inclusions with a mitochondrial membrane protein TOM20-positive edge were frequently observed and clustered into Lewy bodies [81].

### 3.5. DJ-1

Mutations in the protein DJ-1, encoded by the PARK7 gene, cause early-onset autosomal recessive forms of PD with dystonia and psychiatric symptoms [12,82], and oxidized DJ-1 was found in the brains of idiopathic PD individuals [83,84]. DJ-1 is known to coordinate oxidant defenses because its deletion leads to elevated mitochondrial oxidative stress. In human homozygous DJ-1 mutant dopaminergic neurons, mitochondrial oxidant stress was elevated, and neuromelanin and oxidized dopamine progressively accumulated, eventually leading to lysosomal dysfunction. Mitochondrial antioxidants and calcium modulators attenuated the toxic cascade in DJ-1 mutant dopaminergic neurons [85]. DJ-1 also has a role in maintaining mitochondrial function. A study has revealed that DJ-1 is a downstream mediator in PINK1/parkin-dependent mitophagy. DJ-1 loss does not impede the activation of PINK1 or parkin following mitochondrial depolarization but blocks mitophagy by hindering recruitment of the selective autophagy receptor optineurin to the damaged mitochondria. Translocation of DJ-1 to depolarized mitochondria to a close proximity to optineurin is dependent on PINK1 and parkin. Being the downstream molecule, overexpression of DJ-1 failed to rescue the mitophagy defect of PINK1- or parkin-deficient cells [86]. Primary cortical neurons and mouse embryonic fibroblasts with DJ-1 deficiency and lymphoblast cells derived from DJ-1 patients display aberrant mitochondrial morphology, dynamics and ROS production and increased sensitivity to oxidative stress-induced cell death. The fragmented mitochondrial phenotype can be rescued by the expression of PINK1 and parkin [87]. DJ-1 null dopaminergic neurons isolated from mice exhibit a defect in the assembly of complex I, which decreases mitochondrial respiratory chain function. Similarly, fissional/fragmental changes in mitochondrial structure were also observed in these DJ-1 null cells. Overexpression of DJ-1 could reverse the assembly defects as well as the structural and functional abnormalities in DJ-1 null cells [88].

### 3.6. CHCHD2

A recent whole-genome analysis in a Japanese family with late-onset autosomal dominant PD identified missense mutation in the coiled-coil-helix-coiled-coil-helix domain 2 (*CHCHD2*) gene. Clinical features of the patients are typical symptoms of PD such as tremor, bradykinesia, rigidity, postural instability and a good response to L-Dopa treatment [89]. A new *CHCHD2* variant has been reported in a young Caucasian patient with early-onset autosomal recessive PD. Patient fibroblasts carrying this mutation showed a fragmented mitochondrial morphology and reduced oxidative phosphorylation [90]. CHCHD2 is a bi-organellar regulator of mitochondrial function. It is predominantly located in mitochondria under normal conditions and is translocated to the nucleus under stress. In mitochondria, CHCHD2 binds to cytochrome c oxidase (COX), and this binding optimizes COX activity in the process of respiration. A decreased CHCHD2 level in human fibroblasts results in widespread mitochondrial dysfunction, including reduced COX activity and membrane potential, increased reactive oxygen species and mitochondrial fragmentation. In the nucleus, CHCHD2 activates transcription by binding to the conserved oxygen-responsive promoter element of a number of genes involved in stress-responsive pathways including COX and also to its own promoter [91]. It has also been reported that CHCHD2 inhibits apoptosis by binding to Bcl-xL and preventing the accumulation and oligomerization of Bax on the mitochondrial membrane [92].

In the cell model, PD-linked *CHCHD2* mutations impair the binding to CHCHD10 and disrupt the integrity of the MICOS complex, which maintains the mitochondria cristae, eventually leading to mitochondria dysfunction [93]. In Drosophila, CHCHD2 deficiency causes abnormal matrix structures and diminished oxygen respiration in mitochondria, increased oxidative stress, dopaminergic neuron loss and motor dysfunction with age [94]. Similarly, a knock-in mouse model of CHCHD2 p.T61I mutation showed accelerated mortality, progressive motor deficits, dopaminergic neurons loss with age, accompanied by the neuropathological feature of PD, accumulation and aggregation of αSyn and pSyn in the brains. The mitochondria of mouse brains and iPSCs-derived dopaminergic neurons carrying the CHCHD2 p.T61I mutation exhibited aberrant morphology and impaired function. Proteomic and RNA sequencing analysis showed that p.T61I mutation causes mitochondrial dysfunction possibly through repression of insulin-degrading enzyme expression [95].

### 3.7. VPS13C

Whole-genome studies in individuals with early-onset parkinsonism revealed that mutations in vacuolar protein sorting 13C (*VPS13C*) are associated with a distinct form of autosomal recessive early-onset form of PD characterized by rapid and severe disease progression, early cognitive decline and pathological feature resembling diffuse Lewy body disease [96,97,98]. Studies using human cell models showed that VPS13C is located on early endosomes, the Golgi apparatus, the endoplasmic reticulum and the outer mitochondrial membrane. Silencing of VPS13C resulted in mitochondria redistribution to perinuclear region, mitochondrial fragmentation, reduced mitochondrial membrane potential, increased respiration and PINK1/parkin-dependent mitophagy and transcriptional upregulation of PARK2 in response to mitochondrial damage [97].

## 4. Extracellular Mitochondria in Neurological Diseases

Mitochondrial quality control, which is fundamental for maintaining cellular homeostasis, has been thought to be achieved solely through mitophagy. Numerous studies have shown that mitochondria can be transferred out of cells into the extracellular space and can cross cell boundaries, challenging the existing concept [99]. The first description of intercellular organelle transport revealed that highly sensitive nanotubular structures can form de novo between cells, creating complex networks facilitating the selective transfer of membrane vesicles and organelles [100]. Mitochondria or mitochondrial DNA (mtDNA) can move between cells [19]. Extensive mitochondrial transfer from mesenchymal stem cells (MSCs) to macrophages occurred partially through tunneling nanotube-like structures and enhanced macrophage phagocytosis [101]. MSCs also use extracellular vesicles to outsource mitophagy to macrophages [102]. Active transfer from adult stem cells and somatic cells can rescue aerobic respiration in mammalian cells with nonfunctional mitochondria [19]. Most of these studies showed that MSCs are good sources for mitochondria transfer, which provides potential benefits.

**Figure 1:**
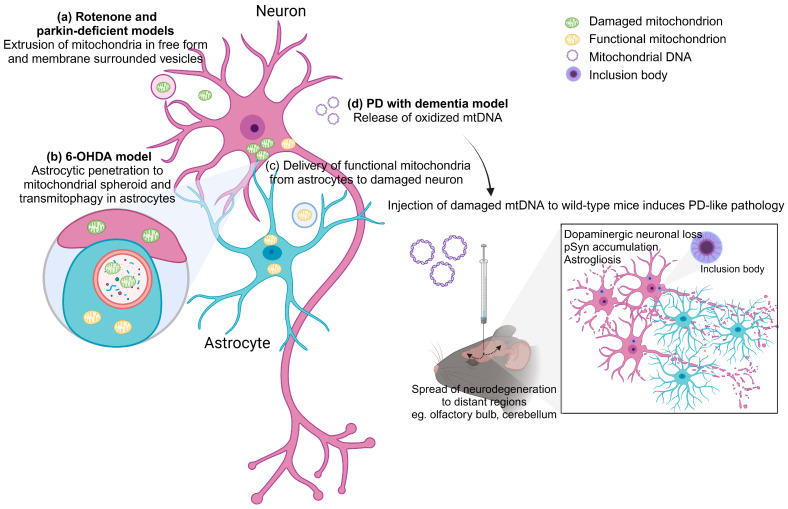
Extracellular mitochondria in PD. (a) In rotenone-induced mitochondrial impairment and parkin-deficient models, damaged mitochondria were extruded from cells in free form or membrane-surrounded vesicles. (b) In a 6-OHDA rodent model of PD, dopaminergic neurons showed accumulation of damaged mitochondria in spheroid structures. These spheroids were penetrated by astrocytic processes, and the mitochondria were transferred to astrocytes and degraded through mitophagy. (c) Neurons can release damaged mitochondria to be internalized by adjacent astrocytes for clearance. On the contrary, astrocytes can release functional mitochondria that enter neurons. (d) In a mouse model of PD with dementia, oxidized mtDNA could be observed being released outside the neurons. Injection of damaged mtDNA into the wild-type mouse brain triggers PD-like pathology including dopaminergic neuronal loss, pSyn accumulation and astrogliosis in the lesioned site. Damaged mtDNA also spread neurodegeneration to distant brain regions. The figure was created with BioRender.com, accessed on 28 November 2023.

Importantly, this phenomenon of mitochondrial transfer also takes place in neuron-glial network (Figure 1). Adult neurons from Caenorhabditis elegans extrude large membrane-surrounded vesicles called exophers that can contain protein aggregates and organelles including mitochondria, with compromised ones preferentially extruded. The extruded exopher transits the hypodermal tissue to be released into the pseudocoelomic fluid, from which materials can later be taken up by distant scavenger cells [103]. Retinal ganglion cell axons of mice were found to shed mitochondria at the optic nerve head to be internalized and degraded by adjacent astrocytes through a process called transmitophagy [104]. In a PD rodent model with intraventricular injection of 6-hydroxydopamine, dopaminergic terminals closest to the lateral ventricle showed an axonal fragmentation and an accumulation of damaged mitochondria in spheroid-like structures. These spheroids were penetrated by astrocytic processes, and the mitochondria were transferred to astrocytes and degraded through mitophagy [105]. Neurons can release damaged mitochondria to be taken up by adjacent astrocytes for clearance and recycling. In an opposite way, astrocytes in mice can release functional mitochondria that enter neurons, mediated by a calcium-dependent mechanism involving CD38 and cyclic ADP ribose signaling. Transient focal cerebral ischemia in mice induced entry of astrocytic mitochondria into adjacent neurons, and this entry amplified cell survival signals [15].

Extracellular mitochondria can be in free form, encapsulated by vesicles or circulating mtDNA (Figure 1). Numerous studies have shown that extracellular mitochondrial functionality reflects intracellular metabolism and underlying tissue metabolic integrity. In a rat model of subarachnoid hemorrhage (SAH), extracellular mitochondria in free form and vesicle structure were detected in cerebrospinal fluid (CSF) at 24 and 72 h after injury. Mitochondrial membrane potentials in CSF were lower in SAH rats compared with sham-operated controls. Extracellular mitochondria were also detected in human CSF samples, and mitochondrial membrane potential levels were reduced after SAH. Higher mitochondrial membrane potentials in the CSF were associated with better clinical recovery at 3 months after SAH onset [16]. In a cultured cell line, mitochondrial quality impairment induced by mitochondrial toxins rotenone and carbonyl cyanide m-chlorophenylhydrazone promotes the extracellular release of predominantly depolarized mitochondria in free form. Parkin- and autophagy-deficient cell lines show increased release of extracellular mitochondria, implying that disruption of mitophagy pathway triggers mitochondria expulsion. Mitochondrial proteins are also present in mouse sera. Higher levels of mitochondrial protein can be detected in the sera of parkin-deficient mice compared to those of wild-type mice. Importantly, fibroblasts and CSF samples derived from PD patients carrying loss-of-function parkin mutations show increased extracellular mitochondria compared to control subjects [14].

Another different form of extracellular mitochondria, cell-free mtDNA (ccf-mtDNA), is a damage-associated molecular pattern acting as a “danger signal”. It has gained interest as a trigger of inflammation due to its similarity to bacterial DNA, its ability to stimulate innate immune responses through Toll-like receptor 9 and its ability to provoke tissue injury when administered in animal models [106,107,108]. A recent study showed that in a spontaneous mouse model of PD with dementia (PDD) caused by deletion of neuronal IFNβ/IFNAR, oxidization, mutation and deletion in mtDNA, which is subsequently released outside the neurons, could be observed. Injection of damaged mtDNA into the mouse brain recreated the clinical and pathological manifestation of PDD, including anxiety as well as motor and cognitive impairments. This is associated with dopaminergic neuronal loss, accumulation of pSyn, oxidative stress and astrogliosis in the injected area. Damaged mtDNA also spread neurodegeneration to brain regions distant from the injection site [8]. The amount of mtDNA generally reflects inflammation status, and it is expected that ccf-mtDNA would be elevated in injuries and diseases. Indeed, patients with anti-NMDAR encephalitis showed higher ccf-mtDNA levels in the CSF compared with controls. There were positive correlations between the CSF levels of ccf-mtDNA and mRS scores which measure the degree of disability of patients with anti-NMDAR encephalitis at both their admission and 6-month follow up [109]. Higher levels of mtDNA were also found in patients with relapsing–remitting multiple sclerosis compared to controls, and there was an inverse correlation between disease duration and mtDNA concentration, suggesting that mitochondria can be involved early in multiple sclerosis [110]. In Alzheimer’s disease (AD), prior studies have showed varying trends with respect to levels of ccf-mtDNA in the CSF of patients [111,112]. An earlier study reported that symptomatic patients at risk of AD and symptomatic AD patients exhibit a significant decrease in circulating ccf-mtDNA in the CSF [111]. On the contrary, the latter demonstrated higher mtDNA counts in the AD group but also a significant degree of interindividual variability within and between phenotypes, which could explain the observed differences between studies [112]. Paradoxically, in the case of PD, there is a significant reduction in ccf-mtDNA in the CSF of patients when compared to controls [17]. However, many factors are likely to influence ccf-mtDNA levels, such as treatment commencement, type and duration as well as comorbidities. This limits the use of ccf-mtDNA as a biomarker of neurodegenerative diseases. Considering inconsistencies in reported disease associations with ccf-mtDNA, studies of ccf-mtDNA where treatment parameters and comorbidity have been excluded or were unavailable should be prudently interpreted. Further studies in independent cohorts are needed to assess the role of ccf-mtDNA in the etiology of PD [113].

## 5. Mitochondrial Transfer in PD

Given the fact that transfer of functional mitochondria induced elevation of mitochondrial membrane potential, increased respiration and improved energy metabolism in recipient cells, healthy mitochondrial transplants to the damaged brain area have been extensively explored as an alternative therapeutic approach.

**Figure 2:**
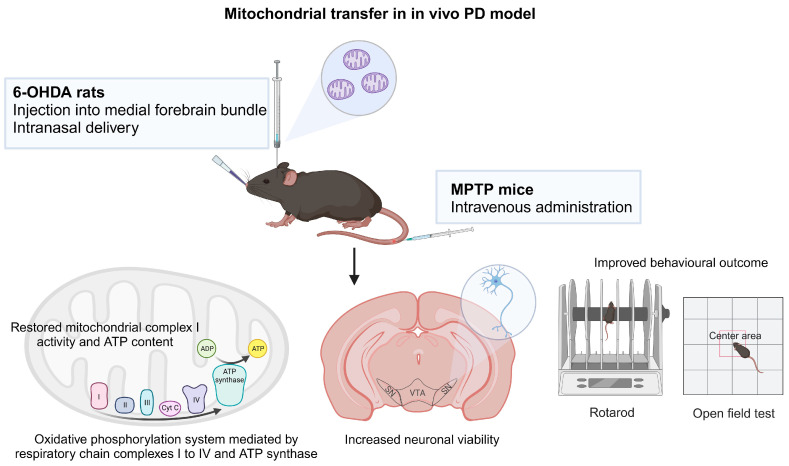
Mitochondrial transfer in PD. Mitochondrial treatment via intravenous administration in experimental MPTP mouse model and intranasal delivery and injection into medial forebrain bundle in 6-OHDA rat model resulted in mitochondrial function recovery, improved neuronal survival and better behavioral outcomes. The figure was created with BioRender.com, accessed on 30 November 2023.

A study showed that the human neuroblastoma SH-SY5Y cell line could naturally be penetrated by mitochondria derived from human hepatoma cells and that when the mitochondria were administered intravenously into mice, all the mice survived with no significant abnormalities. The exogenous mitochondria are distributed ubiquitously in various tissues including brain, liver, kidney, muscle and heart. In normal mice, mitochondrial supplementation enhanced their endurance via a surge of energy production in a forced swimming test. In experimental MPTP-induced PD model mice, mitochondrial treatment improved the locomotor behavior in comparison with the vehicle group by increase of mitochondrial complex I activity and ATP content and suppression of oxidative stress (Figure 2) [114].

Another study demonstrated peptide-mediated mitochondrial delivery in a 6-OHDA-induced PD rat model. In 6-OHDA-administered rats, Pep1-labeled mitochondria injected into the medial forebrain bundle translocated to the SN neurons, restored mitochondrial complex 1 protein and mitochondrial dynamics and thereby ameliorated oxidative DNA damage, resulting in a decrease in dopaminergic neuron loss in the substantia nigra and an improvement in the locomotor activity [115]. The same group later evaluated the feasibility of intranasal mitochondria delivery in unilaterally 6-OHDA lesioned rats and observed mitochondria internalization into the nigral dopaminergic neurons innervating striatum via rostral migratory stream and migration to dopaminergic neurons of the contralateral striatum via interhemispheric commissures. Intranasal infusion of mitochondria led to mitochondrial function recovery and diminished oxidative damage in the lesioned site, hence improved neuronal viability and behavioral outcomes [116]. This study demonstrates that delivering mitochondria without modifications from the nose to the brain is a feasible strategy and is much safer than invasive brain injection. It is less effective than direct injection in functionally restoring mitochondria to dopaminergic neurons, but neuronal survival and behavioral improvement are comparable (Figure 2).

Autologous transplantation of healthy purified mitochondria has been shown to mitigate phenotypes in in vitro and in vivo PD models. However, there are significant technical difficulties in obtaining sufficient quantities of purified functional mitochondria. Furthermore, the storage and maintenance of the mitochondria is particularly challenging as the half-life of mitochondria is short, ranging from days to weeks. A continuous source of healthy mitochondria via intercellular mitochondrial transfer offers an appealing option for therapeutic use. A study showed that iPSC-derived astrocytes can serve as donor to provide functional mitochondria and reverse dopaminergic neurodegeneration and axonal pruning following rotenone exposure in an in vitro model of PD. The use of iPSC-derived astrocytes as mitochondria donors can be further explored for cellular therapy for PD [117].

Taken together, a strategy of mitochondrial replacement would provide an essential and innovative approach to prevent or delay neurodegeneration in PD.

## 6. Conclusions

Mitochondrial dysfunction, whether as a cause or consequence, plays a central and multifaceted role in the pathogenesis of PD. It is an ideal therapeutic target given that modulation of its activity will indeed have an impact on disease progression. However, few successes have been achieved so far for clinical trials employing drugs targeting mitochondrial pathways in PD, possibly due to oversights regarding the multiple interrelated systems and pathways involved in neuronal homeostasis and difficulty in delivering drugs to the site of action [118]. Over the last decade, mitochondrial transplantation has attracted much attention as a novel strategy to replace nonfunctional mitochondria to treat various diseases. As supported by the in vitro and in vivo evidence, replenishing healthy mitochondria to the dopaminergic neurons in degeneration could help revert the disease. However, this intervention might not be suitable in patients with advanced disease progression due to the massive irreversible cell loss. Future therapies in PD will likely rely on combinations of treatments acting on the multiple pathways affected.

## Figures and Tables

**Table 1:** Mitochondrial-function-related familial PD genes.

PARK	Gene	Protein	Form	Clinical Features	Mitochondrial-Related Functions
PARK1 PARK4	*SNCA*	Alpha synuclein (αSyn)	Autosomal dominant	Range from early-onset PD with rapid progression and severe non-motor symptoms including cognitive decline and autonomic dysfunction to classical PD	Contains mitochondrial targeting sequence and can be localized to mitochondria Interacts with complex I and impairs its function Inhibitory function of membrane fusion and causes mitochondria fragmentation when interacting with mitochondrial membrane
PARK2	*PRKN*	Parkin (E3 ubiquitin ligase)	Autosomal recessive	Early-juvenile onset, slow progression, frequent dyskinesia/dystonia	Involved in mitophagy by tagging damaged mitochondria with ubiquitin for autophagic degradation Coordinates mitochondrial lipids to facilitate mitophagy
PARK6	*PINK1*	PTEN-induced putative kinase 1 (mitochondrial serine/threonine protein kinase)	Autosomal recessive	Early onset, slow progression	Initiates mitophagy at a low level by forming phospho-ubiquitin on mitochondria, the signals of which are amplified by parkin to induce robust mitophagy Mitochondrial fission Interacts with and prevents αSyn localization to mitochondria
PARK7	*DJ-1*	Protein/nucleic acid deglycase	Autosomal recessive	Early onset, slow progression, dystonia, psychiatric symptoms	Coordinates oxidant defense against mitochondrial oxidative stress Downstream mediator in PINK1/parkin-dependent mitophagy that recruits autophagy receptors to damaged mitochondria
PARK15	*FBXO7*	F-box protein 7	Autosomal recessive	Early onset with atypical features (pyramidal signs)	Involved in parkin recruitment to damaged mitochondria, mitofusin ubiquitination and mitophagy
PARK22	CHCHD2	Coiled-coil-helix-coiled-coil-helix domain 2	Autosomal recessive/ dominant	Early onset/late onset	Regulation of oxidative phosphorylation by binding to cytochrome c oxidase and optimizing its activity Preserves integrity of the MICOS complex, which maintains mitochondria cristae Inhibition of apoptosis
PARK23	*VPS13C*	Vacuolar protein sorting-associated protein 13C	Autosomal recessive	Early onset, rapid progression, early cognitive decline, widely distributed Lewy bodies	Involved in regulation of PINK1/parkin-mediated mitophagy in response to mitochondrial depolarization

## Data Availability

Not applicable.

## References

[1] Perier C., Vila M. (2012). Mitochondrial Biology and Parkinson’s Disease. Cold Spring Harb. Perspect. Med..

[2] Camandola S., Mattson M.P. (2017). Brain Metabolism in Health, Aging, and Neurodegeneration. EMBO J..

[3] Schapira A.H.V., Cooper J.M., Dexter D., Clark J.B., Jenner P., Marsden C.D. (1990). Mitochondrial Complex I Deficiency in Parkinson’s Disease. J. Neurochem..

[4] Keeney P.M., Xie J., Capaldi R.A., Bennett J.P. (2006). Parkinson’s Disease Brain Mitochondrial Complex I Has Oxidatively Damaged Subunits and Is Functionally Impaired and Misassembled. J. Neurosci..

[5] Bender A., Krishnan K.J., Morris C.M., Taylor G.A., Reeve A.K., Perry R.H., Jaros E., Hersheson J.S., Betts J., Klopstock T. (2006). High Levels of Mitochondrial DNA Deletions in Substantia Nigra Neurons in Aging and Parkinson Disease. Nat. Genet..

[6] Kraytsberg Y., Kudryavtseva E., McKee A.C., Geula C., Kowall N.W., Khrapko K. (2006). Mitochondrial DNA Deletions Are Abundant and Cause Functional Impairment in Aged Human Substantia Nigra Neurons. Nat. Genet..

[7] Nido G.S., Dölle C., Flønes I., Tuppen H.A., Alves G., Tysnes O.B., Haugarvoll K., Tzoulis C. (2018). Ultradeep Mapping of Neuronal Mitochondrial Deletions in Parkinson’s Disease. Neurobiol. Aging.

[8] Tresse E., Marturia-Navarro J., Qi W., Sew G., Cisquella-Serra M., Jaberi E., Riera-Ponsati L., Fauerby N., Hu E., Kretz O. (2023). Mitochondrial DNA Damage Triggers Spread of Parkinson’s Disease-like Pathology. Mol. Psychiatry.

[9] Reeve A., Meagher M., Lax N., Simcox E., Hepplewhite P., Jaros E., Turnbull D. (2013). The Impact of Pathogenic Mitochondrial DNA Mutations on Substantia Nigra Neurons. J. Neurosci..

[10] Kitada T., Asakawa S., Hattori N., Matsumine H., Yamamura Y., Minoshima S., Yokochi M., Mizuno Y., Shimizu N. (1998). Mutations in the Parkin Gene Cause Autosomal Recessive Juvenile Parkinsonism. Nature.

[11] Valente E.M., Abou-Sleiman P.M., Caputo V., Muqit M.M.K., Harvey K., Gispert S., Ali Z., Del Turco D., Bentivoglio A.R., Healy D.G. (2004). Hereditary Early-Onset Parkinson’s Disease Caused by Mutations in PINK1. Science.

[12] Bonifati V., Rizzu P., Van Baren M.J., Schaap O., Breedveld G.J., Krieger E., Dekker M.C.J., Squitieri F., Ibanez P., Joosse M. (2003). Mutations in the DJ-1 Gene Associated with Autosomal Recessive Early-Onset Parkinsonism. Science.

[13] Karimi-Moghadam A., Charsouei S., Bell B., Jabalameli M.R. (2018). Parkinson Disease from Mendelian Forms to Genetic Susceptibility: New Molecular Insights into the Neurodegeneration Process. Cell. Mol. Neurobiol..

[14] Choong C.J., Okuno T., Ikenaka K., Baba K., Hayakawa H., Koike M., Yokota M., Doi J., Kakuda K., Takeuchi T. (2021). Alternative Mitochondrial Quality Control Mediated by Extracellular Release. Autophagy.

[15] Hayakawa K., Esposito E., Wang X., Terasaki Y., Liu Y., Xing C., Ji X., Lo E.H. (2016). Transfer of Mitochondria from Astrocytes to Neurons after Stroke. Nature.

[16] Chou S.H.Y., Lan J., Esposito E., Ning M.M., Balaj L., Ji X., Lo E.H., Hayakawa K. (2017). Extracellular Mitochondria in Cerebrospinal Fluid and Neurological Recovery after Subarachnoid Hemorrhage. Stroke.

[17] Pyle A., Brennan R., Kurzawa-Akanbi M., Yarnall A., Thouin A., Mollenhauer B., Burn D., Chinnery P.F., Hudson G. (2015). Reduced Cerebrospinal Fluid Mitochondrial DNA Is a Biomarker for Early-Stage Parkinson’s Disease. Ann. Neurol..

[18] Jain R., Begum N., Tryphena K.P., Singh S.B., Srivastava S., Rai S.N., Vamanu E., Khatri D.K. (2023). Inter and Intracellular Mitochondrial Transfer: Future of Mitochondrial Transplant Therapy in Parkinson’s Disease. Biomed. Pharmacother..

[19] Spees J.L., Olson S.D., Whitney M.J., Prockop D.J. (2006). Mitochondrial Transfer between Cells Can Rescue Aerobic Respiration. Proc. Natl. Acad. Sci. USA.

[20] Islam M.N., Das S.R., Emin M.T., Wei M., Sun L., Westphalen K., Rowlands D.J., Quadri S.K., Bhattacharya S., Bhattacharya J. (2012). Mitochondrial Transfer from Bone-Marrow–Derived Stromal Cells to Pulmonary Alveoli Protects against Acute Lung Injury. Nat. Med..

[21] William Langston J., Ballard P., Tetrud J.W., Irwin I. (1983). Chronic Parkinsonism in Humans Due to a Product of Meperidine-Analog Synthesis. Science.

[22] Nicklas W.J., Vyas I., Heikkila R.E. (1985). Inhibition of NADH-Linked Oxidation in Brain Mitochondria by 1-Methyl-4-Phenyl-Pyridine, a Metabolite of the Neurotoxin, 1-Methyl-4-Phenyl-1,2,5,6-Tetrahydropyridine. Life Sci..

[23] Ramsay R.R., Salach J.I., Dadgar J., Singer T.P. (1986). Inhibition of Mitochondrial NADH Dehydrogenase by Pyridine Derivatives and Its Possible Relation to Experimental and Idiopathic Parkinsonism. Biochem. Biophys. Res. Commun..

[24] Desai V.G., Feuers R.J., Hart R.W., Ali S.F. (1996). MPP+-Induced Neurotoxicity in Mouse Is Age-Dependent: Evidenced by the Selective Inhibition of Complexes of Electron Transport. Brain Res..

[25] Andreyev A.Y., Kushnareva Y.E., Starkov A.A. (2005). Mitochondrial Metabolism of Reactive Oxygen Species. Biochemistry.

[26] Sherer T.B., Richardson J.R., Testa C.M., Seo B.B., Panov A.V., Yagi T., Matsuno-Yagi A., Miller G.W., Greenamyre J.T. (2007). Mechanism of Toxicity of Pesticides Acting at Complex I: Relevance to Environmental Etiologies of Parkinson’s Disease. J. Neurochem..

[27] Mizuno Y., Ohta S., Tanaka M., Takamiya S., Suzuki K., Sato T., Oya H., Ozawa T., Kagawa Y. (1989). Deficiencies in Complex I Subunits of the Respiratory Chain in Parkinson’s Disease. Biochem. Biophys. Res. Commun..

[28] Parker W.D., Parks J.K., Swerdlow R.H. (2008). Complex I Deficiency in Parkinson’s Disease Frontal Cortex. Brain Res..

[29] Haas R.H., Nasirian F., Nakano K., Ward D., Pay M., Hill R., Shults C.W. (1995). Low Platelet Mitochondrial Complex I and Complex II/III Activity in Early Untreated Parkinson’s Disease. Ann. Neurol..

[30] Bindoff L.A., Birch-Machin M.A., Cartlidge N.E.F., Parker W.D., Turnbull D.M. (1991). Respiratory Chain Abnormalities in Skeletal Muscle from Patients with Parkinson’s Disease. J. Neurol. Sci..

[31] González-Rodríguez P., Zampese E., Stout K.A., Guzman J.N., Ilijic E., Yang B., Tkatch T., Stavarache M.A., Wokosin D.L., Gao L. (2021). Disruption of Mitochondrial Complex I Induces Progressive Parkinsonism. Nature.

[32] Billingsley K.J., Barbosa I.A., Bandrés-Ciga S., Quinn J.P., Bubb V.J., Deshpande C., Botia J.A., Reynolds R.H., Zhang D., Simpson M.A. (2019). Mitochondria Function Associated Genes Contribute to Parkinson’s Disease Risk and Later Age at Onset. NPJ Park. Dis..

[33] Shimura H., Hattori N., Kubo S.I., Mizuno Y., Asakawa S., Minoshima S., Shimizu N., Iwai K., Chiba T., Tanaka K. (2000). Familial Parkinson Disease Gene Product, Parkin, Is a Ubiquitin-Protein Ligase. Nat. Genet..

[34] Lin C.-C., Yan J., Kapur M.D., Norris K.L., Hsieh C.-W., Huang D., Vitale N., Lim K.-L., Guan Z., Wang X.-F. (2022). Parkin Coordinates Mitochondrial Lipid Remodeling to Execute Mitophagy. EMBO Rep..

[35] Ardah M.T., Radwan N., Khan E., Kitada T., Haque M.E. (2023). Parkin Precipitates on Mitochondria via Aggregation and Autoubiquitination. Int. J. Mol. Sci..

[36] Machida Y., Chiba T., Takayanagi A., Tanaka Y., Asanuma M., Ogawa N., Koyama A., Iwatsubo T., Ito S., Jansen P.H. (2005). Common Anti-Apoptotic Roles of Parkin and α-Synuclein in Human Dopaminergic Cells. Biochem. Biophys. Res. Commun..

[37] Palacino J.J., Sagi D., Goldberg M.S., Krauss S., Motz C., Wacker M., Klose J., Shen J. (2004). Mitochondrial Dysfunction and Oxidative Damage in Parkin-Deficient Mice. J. Biol. Chem..

[38] Pesah Y., Pham T., Burgess H., Middlebrooks B., Verstreken P., Zhou Y., Harding M., Bellen H., Mardon G. (2004). Drosophila Parkin Mutants Have Decreased Mass and Cell Size and Increased Sensitivity to Oxygen Radical Stress. Development.

[39] Greene J.C., Whitworth A.J., Kuo I., Andrews L.A., Feany M.B., Pallanck L.J. (2003). Mitochondrial Pathology and Apoptotic Muscle Degeneration in *Drosophila parkin* Mutants. Proc. Natl. Acad. Sci. USA.

[40] Imaizumi Y., Okada Y., Akamatsu W., Koike M., Kuzumaki N., Hayakawa H., Nihira T., Kobayashi T., Ohyama M., Sato S. (2012). Mitochondrial Dysfunction Associated with Increased Oxidative Stress and α-Synuclein Accumulation in PARK2 IPSC-Derived Neurons and Postmortem Brain Tissue. Mol. Brain.

[41] Shin J.H., Ko H.S., Kang H., Lee Y., Lee Y.I., Pletinkova O., Troconso J.C., Dawson V.L., Dawson T.M. (2011). PARIS (ZNF746) Repression of PGC-1α Contributes to Neurodegeneration in Parkinson’s Disease. Cell.

[42] Stevens D.A., Lee Y., Kang H.C., Lee B.D., Lee Y.I., Bower A., Jiang H., Kang S.U., Andrabi S.A., Dawson V.L. (2015). Parkin Loss Leads to Paris-Dependent Declines in Mitochondrial Mass and Respiration. Proc. Natl. Acad. Sci. USA.

[43] Chung K.K.K., Thomas B., Li X., Pletnikova O., Troncoso J.C., Marsh L., Dawson V.L., Dawson T.M. (2004). S-Nitrosylation of Parkin Regulates Ubiquitination and Compromises Parkin’s Protective Function. Science.

[44] Yao D., Gu Z., Nakamura T., Shi Z.Q., Ma Y., Gaston B., Palmer L.A., Rockenstein E.M., Zhang Z., Masliah E. (2004). Nitrosative Stress Linked to Sporadic Parkinson’s Disease: S-Nitrosylation of Parkin Regulates Its E3 Ubiquitin Ligase Activity. Proc. Natl. Acad. Sci. USA.

[45] LaVoie M.J., Ostaszewski B.L., Weihofen A., Schlossmacher M.G., Selkoe D.J. (2005). Dopamine Covalently Modifies and Functionally Inactivates Parkin. Nat. Med..

[46] Vandiver M.S., Paul B.D., Xu R., Karuppagounder S., Rao F., Snowman A.M., Seok Ko H., Il Lee Y., Dawson V.L., Dawson T.M. (2013). Sulfhydration Mediates Neuroprotective Actions of Parkin. Nat. Commun..

[47] Jin S.M., Lazarou M., Wang C., Kane L.A., Narendra D.P., Youle R.J. (2010). Mitochondrial Membrane Potential Regulates PINK1 Import and Proteolytic Destabilization by PARL. J. Cell Biol..

[48] Matsuda N., Sato S., Shiba K., Okatsu K., Saisho K., Gautier C.A., Sou Y.S., Saiki S., Kawajiri S., Sato F. (2010). PINK1 Stabilized by Mitochondrial Depolarization Recruits Parkin to Damaged Mitochondria and Activates Latent Parkin for Mitophagy. J. Cell Biol..

[49] Yamano K., Youle R.J. (2013). PINK1 Is Degraded through the N-End Rule Pathway. Autophagy.

[50] Narendra D.P., Jin S.M., Tanaka A., Suen D.F., Gautier C.A., Shen J., Cookson M.R., Youle R.J. (2010). PINK1 Is Selectively Stabilized on Impaired Mitochondria to Activate Parkin. PLoS Biol..

[51] Vives-Bauza C., Zhou C., Huang Y., Cui M., De Vries R.L.A., Kim J., May J., Tocilescu M.A., Liu W., Ko H.S. (2010). PINK1-Dependent Recruitment of Parkin to Mitochondria in Mitophagy. Proc. Natl. Acad. Sci. USA.

[52] Koyano F., Okatsu K., Kosako H., Tamura Y., Go E., Kimura M., Kimura Y., Tsuchiya H., Yoshihara H., Hirokawa T. (2014). Ubiquitin Is Phosphorylated by PINK1 to Activate Parkin. Nature.

[53] Lazarou M., Sliter D.A., Kane L.A., Sarraf S.A., Wang C., Burman J.L., Sideris D.P., Fogel A.I., Youle R.J. (2015). The Ubiquitin Kinase PINK1 Recruits Autophagy Receptors to Induce Mitophagy. Nature.

[54] Wang H.L., Chou A.H., Yeh T.H., Li A.H., Chen Y.L., Kuo Y.L., Tsai S.R., Yu S.T. (2007). PINK1 Mutants Associated with Recessive Parkinson’s Disease Are Defective in Inhibiting Mitochondrial Release of Cytochrome c. Neurobiol. Dis..

[55] Zhou Z.D., Refai F.S., Xie S.P., Ng S.H., Chan C.H.S., Ho P.G.H., Zhang X.D., Lim T.M., Tan E.K. (2014). Mutant PINK1 Upregulates Tyrosine Hydroxylase and Dopamine Levels, Leading to Vulnerability of Dopaminergic Neurons. Free Radic. Biol. Med..

[56] Clark I.E., Dodson M.W., Jiang C., Cao J.H., Huh J.R., Seol J.H., Yoo S.J., Hay B.A., Guo M. (2006). Drosophila Pink1 Is Required for Mitochondrial Function and Interacts Genetically with Parkin. Nature.

[57] Wang H.L., Chou A.H., Wu A.S., Chen S.Y., Weng Y.H., Kao Y.C., Yeh T.H., Chu P.J., Lu C.S. (2011). PARK6 PINK1 Mutants Are Defective in Maintaining Mitochondrial Membrane Potential and Inhibiting ROS Formation of Substantia Nigra Dopaminergic Neurons. Biochim. Biophys. Acta (BBA)—Mol. Basis Dis..

[58] Gautier C.A., Kitada T., Shen J. (2008). Loss of PINK1 Causes Mitochondrial Functional Defects and Increased Sensitivity to Oxidative Stress. Proc. Natl. Acad. Sci. USA.

[59] Yang Y., Ouyang Y., Yang L., Beal M.F., McQuibban A., Vogel H., Lu B. (2008). Pink1 Regulates Mitochondrial Dynamics through Interaction with the Fission/Fusion Machinery. Proc. Natl. Acad. Sci. USA.

[60] Lee M.J., Chien W.L., Lee T.R., Hung S.Y., Kang K.H., Fu W.M. (2011). Impairment of Oxidative Stress-Induced Heme Oxygenase-1 Expression by the Defect of Parkinson-Related Gene of PINK1. J. Neurochem..

[61] Moisoi N., Fedele V., Edwards J., Martins L.M. (2014). Loss of PINK1 Enhances Neurodegeneration in a Mouse Model of Parkinson’s Disease Triggered by Mitochondrial Stress. Neuropharmacology.

[62] Oliveras-Salvá M., Macchi F., Coessens V., Deleersnijder A., Gérard M., Van der Perren A., Van den Haute C., Baekelandt V. (2014). Alpha-Synuclein-Induced Neurodegeneration Is Exacerbated in PINK1 Knockout Mice. Neurobiol. Aging.

[63] Gispert S., Brehm N., Weil J., Seidel K., Rüb U., Kern B., Walter M., Roeper J., Auburger G. (2015). Potentiation of Neurotoxicity in Double-Mutant Mice with Pink1 Ablation and A53T-SNCA Overexpression. Hum. Mol. Genet..

[64] Liu J., Wang X., Lu Y., Duan C., Gao G., Lu L., Yang H. (2017). Pink1 Interacts with α-Synuclein and Abrogates α-Synuclein-Induced Neurotoxicity by Activating Autophagy. Cell Death Dis..

[65] Todd A.M., Staveley B.E. (2008). Pink1 Suppresses α-Synuclein-Induced Phenotypes in a Drosophila Model of Parkinson’s Disease. Genome.

[66] Todd A.M., Staveley B.E. (2012). Expression of Pink1 with α-Synuclein in the Dopaminergic Neurons of Drosophila Leads to Increases in Both Lifespan and Healthspan. Genet. Mol. Res..

[67] Burchell V.S., Nelson D.E., Sanchez-Martinez A., Delgado-Camprubi M., Ivatt R.M., Pogson J.H., Randle S.J., Wray S., Lewis P.A., Houlden H. (2013). The Parkinson’s Disease–Linked Proteins Fbxo7 and Parkin Interact to Mediate Mitophagy. Nat. Neurosci..

[68] Fonzo A.D., Dekker M.C.J., Montagna P., Baruzzi A., Yonova E.H., Guedes L.C., Szczerbinska A., Zhao T., Dubbel-Hulsman L.O.M., Wouters C.H. (2009). FBXO7 Mutations Cause Autosomal Recessive, Early-Onset Parkinsonian-Pyramidal Syndrome. Neurology.

[69] Sanchez-Martinez Id A., Martinez A., Whitworth Id A.J. (2023). FBXO7/Ntc and USP30 Antagonistically Set the Ubiquitination Threshold for Basal Mitophagy and Provide a Target for Pink1 Phosphorylation in Vivo. PLoS Biol..

[70] Kraus F., Goodall E.A., Smith I.R., Jiang Y., Paoli J.C., Adolf F., Zhang J., Paulo J.A., Schulman B.A., Harper J.W. (2023). PARK15/FBXO7 Is Dispensable for PINK1/Parkin Mitophagy in INeurons and HeLa Cell Systems. EMBO Rep..

[71] Devi L., Raghavendran V., Prabhu B.M., Avadhani N.G., Anandatheerthavarada H.K. (2008). Mitochondrial Import and Accumulation of α-Synuclein Impair Complex I in Human Dopaminergic Neuronal Cultures and Parkinson Disease Brain. J. Biol. Chem..

[72] Chinta S.J., Mallajosyula J.K., Rane A., Andersen J.K. (2010). Mitochondrial Alpha-Synuclein Accumulation Impairs Complex I Function in Dopaminergic Neurons and Results in Increased Mitophagy in Vivo. Neurosci. Lett..

[73] Liu G., Zhang C., Yin J., Li X., Cheng F., Li Y., Yang H., Uéda K., Chan P., Yu S. (2009). α-Synuclein Is Differentially Expressed in Mitochondria from Different Rat Brain Regions and Dose-Dependently down-Regulates Complex I Activity. Neurosci. Lett..

[74] Hsu L.J., Sagara Y., Arroyo A., Rockenstein E., Sisk A., Mallory M., Wong J., Takenouchi T., Hashimoto M., Masliah E. (2000). α-Synuclein Promotes Mitochondrial Deficit and Oxidative Stress. Am. J. Pathol..

[75] Kamp F., Exner N., Lutz A.K., Wender N., Hegermann J., Brunner B., Nuscher B., Bartels T., Giese A., Beyer K. (2010). Inhibition of Mitochondrial Fusion by α-Synuclein Is Rescued by PINK1, Parkin and DJ-1. EMBO J..

[76] Nakamura K., Nemani V.M., Azarbal F., Skibinski G., Levy J.M., Egami K., Munishkina L., Zhang J., Gardner B., Wakabayashi J. (2011). Direct Membrane Association Drives Mitochondrial Fission by the Parkinson Disease-Associated Protein α-Synuclein. J. Biol. Chem..

[77] Cole N.B., DiEuliis D., Leo P., Mitchell D.C., Nussbaum R.L. (2008). Mitochondrial Translocation of α-Synuclein Is Promoted by Intracellular Acidification. Exp. Cell Res..

[78] Choi M.L., Chappard A., Singh B.P., Maclachlan C., Rodrigues M., Fedotova E.I., Berezhnov A.V., De S., Peddie C.J., Athauda D. (2022). Pathological Structural Conversion of α-Synuclein at the Mitochondria Induces Neuronal Toxicity. Nat. Neurosci..

[79] Choubey V., Safiulina D., Vaarmann A., Cagalinec M., Wareski P., Kuum M., Zharkovsky A., Kaasik A. (2011). Mutant A53T α-Synuclein Induces Neuronal Death by Increasing Mitochondrial Autophagy. J. Biol. Chem..

[80] Martin L.J., Pan Y., Price A.C., Sterling W., Copeland N.G., Jenkins N.A., Price D.L., Lee M.K. (2006). Parkinson’s Disease α-Synuclein Transgenic Mice Develop Neuronal Mitochondrial Degeneration and Cell Death. J. Neurosci..

[81] Sumi-Akamaru H., Beck G., Shinzawa K., Kato S., Riku Y., Yoshida M., Fujimura H., Tsujimoto Y., Sakoda S., Mochizuki H. (2016). High Expression of α-Synuclein in Damaged Mitochondria with PLA2G6 Dysfunction. Acta Neuropathol. Commun..

[82] Bras J.M., Guerreiro R.J., Teo J.T.H., Darwent L., Vaughan J., Molloy S., Hardy J., Schneider S.A. (2014). Atypical Parkinsonism-Dystonia Syndrome Caused by a Novel DJ1 Mutation. Mov. Disord. Clin. Pract..

[83] Saito Y. (2014). Oxidized DJ-1 as a Possible Biomarker of Parkinson’s Disease. J. Clin. Biochem. Nutr..

[84] Saito Y., Hamakubo T., Yoshida Y., Ogawa Y., Hara Y., Fujimura H., Imai Y., Iwanari H., Mochizuki Y., Shichiri M. (2009). Preparation and Application of Monoclonal Antibodies against Oxidized DJ-1. Significant Elevation of Oxidized DJ-1 in Erythrocytes of Early-Stage Parkinson Disease Patients. Neurosci. Lett..

[85] Burbulla L.F., Song P., Mazzulli J.R., Zampese E., Wong Y.C., Jeon S., Santos D.P., Blanz J., Obermaier C.D., Strojny C. (2017). Dopamine Oxidation Mediates Mitochondrial and Lysosomal Dysfunction in Parkinson’s Disease. Science.

[86] Imberechts D., Kinnart I., Wauters F., Terbeek J., Manders L., Wierda K., Eggermont K., Madeiro R.F., Sue C., Verfaillie C. (2022). DJ-1 Is an Essential Downstream Mediator in PINK1/Parkin-Dependent. Brain.

[87] Irrcher I., Aleyasin H., Seifert E.L., Hewitt S.J., Chhabra S., Phillips M., Lutz A.K., Rousseaux M.W.C., Bevilacqua L., Jahani-Asl A. (2010). Loss of the Parkinson’s Disease-Linked Gene DJ-1 Perturbs Mitochondrial Dynamics. Hum. Mol. Genet..

[88] Heo J.Y., Park J.H., Kim S.J., Seo K.S., Han J.S., Lee S.H., Kim J.M., Park J.I., Park S.K., Lim K. (2012). DJ-1 Null Dopaminergic Neuronal Cells Exhibit Defects in Mitochondrial Function and Structure: Involvement of Mitochondrial Complex I Assembly. PLoS ONE.

[89] Funayama M., Ohe K., Amo T., Furuya N., Yamaguchi J., Saiki S., Li Y., Ogaki K., Ando M., Yoshino H. (2015). CHCHD2 Mutations in Autosomal Dominant Late-Onset Parkinson’s Disease: A Genome-Wide Linkage and Sequencing Study. Lancet Neurol..

[90] Lee R.G., Sedghi M., Salari M., Shearwood A.-M.J., Stentenbach M., Kariminejad A., Goullee H., Rackham O., Laing N.G., Tajsharghi H. (2018). Early-Onset Parkinson Disease Caused by a Mutation in CHCHD2 and Mitochondrial Dysfunction. Neurol. Genet..

[91] Aras S., Bai M., Lee I., Springett R., Hüttemann M., Grossman L.I. (2015). MNRR1 (Formerly CHCHD2) Is a Bi-Organellar Regulator of Mitochondrial Metabolism. Mitochondrion.

[92] Liu Y., Clegg H.V., Leslie P.L., Di J., Tollini L.A., He Y., Kim T.H., Jin A., Graves L.M., Zheng J. (2014). CHCHD2 Inhibits Apoptosis by Interacting with Bcl-x L to Regulate Bax Activation. Cell Death Differ..

[93] Zhou W., Ma D., Sun A.X., Tran H.-D., Ma D., Singh B.K., Zhou J., Zhang J., Wang D., Zhao Y. (2019). PD-Linked CHCHD2 Mutations Impair CHCHD10 and MICOS Complex Leading to Mitochondria Dysfunction. Hum. Mol. Genet..

[94] Meng H., Yamashita C., Shiba-Fukushima K., Inoshita T., Funayama M., Sato S., Hatta T., Natsume T., Umitsu M., Takagi J. (2017). Loss of Parkinson’s Disease-Associated Protein CHCHD2 Affects Mitochondrial Crista Structure and Destabilizes Cytochrome c. Nat. Commun..

[95] Fan L., Zhang S., Li X., Hu Z., Yang J., Zhang S., Zheng H., Su Y., Luo H., Liu X. (2023). CHCHD2 p.Thr61Ile Knock-in Mice Exhibit Motor Defects and Neuropathological Features of Parkinson’s Disease. Brain Pathol..

[96] Nalls M.A., Pankratz N., Lill C.M., Do C.B., Hernandez D.G., Saad M., Destefano A.L., Kara E., Bras J., Sharma M. (2014). Large-Scale Meta-Analysis of Genome-Wide Association Data Identifies Six New Risk Loci for Parkinson’s Disease. Nat. Genet..

[97] Lesage S., Drouet V., Majounie E., Deramecourt V., Jacoupy M., Nicolas A., Cormier-Dequaire F., Hassoun S.M., Pujol C., Ciura S. (2016). Loss of VPS13C Function in Autosomal-Recessive Parkinsonism Causes Mitochondrial Dysfunction and Increases PINK1/Parkin-Dependent Mitophagy. Am. J. Hum. Genet..

[98] Gu X., Li C., Chen Y., Ou R., Cao B., Wei Q., Hou Y., Zhang L., Song W., Zhao B. (2020). Mutation Screening and Burden Analysis of VPS13C in Chinese Patients with Early-Onset Parkinson’s Disease. Neurobiol. Aging.

[99] Miliotis S., Nicolalde B., Ortega M., Yepez J., Caicedo A. (2019). Forms of Extracellular Mitochondria and Their Impact in Health. Mitochondrion.

[100] Rustom A., Saffrich R., Markovic I., Walther P., Gerdes H.H. (2004). Nanotubular Highways for Intercellular Organelle Transport. Science.

[101] Jackson M.V., Morrison T.J., Doherty D.F., McAuley D.F., Matthay M.A., Kissenpfennig A., O’Kane C.M., Krasnodembskaya A.D. (2016). Mitochondrial Transfer via Tunneling Nanotubes Is an Important Mechanism by Which Mesenchymal Stem Cells Enhance Macrophage Phagocytosis in the In Vitro and In Vivo Models of ARDS. Stem Cells.

[102] Phinney D.G., Di Giuseppe M., Njah J., Sala E., Shiva S., St Croix C.M., Stolz D.B., Watkins S.C., Di Y.P., Leikauf G.D. (2015). Mesenchymal Stem Cells Use Extracellular Vesicles to Outsource Mitophagy and Shuttle MicroRNAs. Nat. Commun..

[103] Melentijevic I., Toth M.L., Arnold M.L., Guasp R.J., Harinath G., Nguyen K.C., Taub D., Parker J.A., Neri C., Gabel C.V. (2017). *C. elegans* Neurons Jettison Protein Aggregates and Mitochondria under Neurotoxic Stress. Nature.

[104] Davis C.H.O., Kim K.Y., Bushong E.A., Mills E.A., Boassa D., Shih T., Kinebuchi M., Phan S., Zhou Y., Bihlmeyer N.A. (2014). Transcellular Degradation of Axonal Mitochondria. Proc. Natl. Acad. Sci. USA.

[105] Morales I., Sanchez A., Puertas-Avendaño R., Rodriguez-Sabate C., Perez-Barreto A., Rodriguez M. (2020). Neuroglial Transmitophagy and Parkinson’s Disease. Glia.

[106] Thurairajah K., Briggs G.D., Balogh Z.J. (2018). The Source of Cell-Free Mitochondrial DNA in Trauma and Potential Therapeutic Strategies. Eur. J. Trauma Emerg. Surg..

[107] Gu X., Wu G., Yao Y., Zeng J., Shi D., Lv T., Luo L., Song Y. (2015). Intratracheal Administration of Mitochondrial DNA Directly Provokes Lung Inflammation through the TLR9-P38 MAPK Pathway. Free Radic. Biol. Med..

[108] Xie L., Liu S., Cheng J., Wang L., Liu J., Gong J. (2017). Exogenous Administration of Mitochondrial DNA Promotes Ischemia Reperfusion Injury via TLR9-P38 MAPK Pathway. Regul. Toxicol. Pharmacol..

[109] Peng Y., Zheng D., Zhang X., Pan S., Ji T., Zhang J., Shen H.Y., Wang H.H. (2019). Cell-Free Mitochondrial DNA in the CSF: A Potential Prognostic Biomarker of Anti-NMDAR Encephalitis. Front. Immunol..

[110] Varhaug K.N., Vedeler C.A., Myhr K.M., Aarseth J.H., Tzoulis C., Bindoff L.A. (2017). Increased Levels of Cell-Free Mitochondrial DNA in the Cerebrospinal Fluid of Patients with Multiple Sclerosis. Mitochondrion.

[111] Podlesniy P., Figueiro-Silva J., Llado A., Antonell A., Sanchez-Valle R., Alcolea D., Lleo A., Molinuevo J.L., Serra N., Trullas R. (2013). Low Cerebrospinal Fluid Concentration of Mitochondrial DNA in Preclinical Alzheimer Disease. Ann. Neurol..

[112] Cervera-Carles L., Alcolea D., Estanga A., Ecay-Torres M., Izagirre A., Clerigué M., García-Sebastián M., Villanúa J., Escalas C., Blesa R. (2017). Cerebrospinal Fluid Mitochondrial DNA in the Alzheimer’s Disease Continuum. Neurobiol. Aging.

[113] Lowes H., Pyle A., Santibanez-Koref M., Hudson G. (2020). Circulating Cell-Free Mitochondrial DNA Levels in Parkinson’s Disease Are Influenced by Treatment. Mol. Neurodegener..

[114] Shi X., Zhao M., Fu C., Fu A. (2017). Intravenous Administration of Mitochondria for Treating Experimental Parkinson’s Disease. Mitochondrion.

[115] Chang J.C., Wu S.L., Liu K.H., Chen Y.H., Chuang C.S., Cheng F.C., Su H.L., Wei Y.H., Kuo S.J., Liu C.S. (2016). Allogeneic/Xenogeneic Transplantation of Peptide-Labeled Mitochondria in Parkinson’s Disease: Restoration of Mitochondria Functions and Attenuation of 6-Hydroxydopamine-Induced Neurotoxicity. Transl. Res..

[116] Chang J.C., Chao Y.C., Chang H.S., Wu Y.L., Chang H.J., Lin Y.S., Cheng W.L., Lin T.T., Liu C.S. (2021). Intranasal Delivery of Mitochondria for Treatment of Parkinson’s Disease Model Rats Lesioned with 6-Hydroxydopamine. Sci. Rep..

[117] Cheng X.Y., Biswas S., Li J., Mao C.J., Chechneva O., Chen J., Li K., Li J., Zhang J.R., Liu C.F. (2020). Human IPSCs Derived Astrocytes Rescue Rotenone-Induced Mitochondrial Dysfunction and Dopaminergic Neurodegeneration in Vitro by Donating Functional Mitochondria. Transl. Neurodegener..

[118] Prasuhn J., Davis R.L., Kumar K.R. (2021). Targeting Mitochondrial Impairment in Parkinson’s Disease: Challenges and Opportunities. Front. Cell Dev. Biol..

